# Treatment with rituximab and brentuximab vedotin in a patient of common variable immune deficiency-associated classic Hodgkin lymphoma

**DOI:** 10.1186/s40364-016-0061-8

**Published:** 2016-03-09

**Authors:** Efren Rael, Kevin Rakszawski, Kristian Koller, Michael Bayerl, Manish Butte, Hong Zheng

**Affiliations:** Department of Allergy and Immunology, Stanford University, School of Medicine, Stanford, CA 94305 USA; Penn State Hershey Cancer Institute, Penn State University College of Medicine, 500 University Drive, P.O. Box 850, Hershey, PA 17033 USA; Department of Pathology, Penn State Hershey Medical Center, Penn State University College of Medicine, Hershey, PA 17033 USA

**Keywords:** Brentuximab, Rituximab, CVID, Hodgkin lymphoma

## Abstract

**Background:**

Patients with common variable immunodeficiency (CVID) have an increased risk of developing lymphoproliferative diseases, including non-Hodgkins lymphoma (Blood 116:1228–1234, 2010; Blood 119:1650–7, 2012). The incidence and prognosis of Hodgkin lymphoma in this population is not clear, with only a few case reports in the literature. Conventional cytotoxic chemotherapy, although highly efficacious in treating Hodgkin lymphoma in immune competent patients, is problematic in patients with CVID due to the increased risk of infectious complications (Ther Umsch 69:687–91, 2012; Pediatr Hematol Oncol 24:337–42, 2012). Rituximab and brentuximab vedotin are both targeted agents used to treat lymphomas that express CD20 and CD30, respectively. Compared to cytotoxic chemotherapy typically used in Hodgkin lymphoma, these agents are better tolerated with minimal side effects. This makes them an attractive option for treating lymphoma in patients who have significant co-morbidities, including those with immune deficiencies. Additionally, rituximab has been used safely to treat autoimmune cytopenias in patients with CVID5. However, the role of these targeted therapies in CVID-associated Hodgkin lymphoma has not been reported.

**Case Presentation:**

Here we report the case of a 25 year old female diagnosed with CVID-associated classic Hodgkin lymphoma, who achieved a complete remission following treatment with rituximab followed by brentuximab vedotin.

**Conclusions:**

We demonstrate that rituximab and brentuximab are likely safe and effective in CVID-associated Hodgkin lymphoma, providing a feasible and potentially optimal treatment option for this patient population.

## Background

Common variable immunodeficiency (CVID) is a rare immune disorder characterized by decreased immunoglobulin production resulting from a variety of gene defects. Clinically, patients with CVID have a diverse phenotype, but can commonly present with recurrent infections, autoimmune disorders, and lymphoproliferative disorders including malignancies [1]. Treatment of CVID consists of lifelong immunoglobulin replacement [2].

The overall incidence of malignant disease appears to be increased in CVID, with a nearly 5-fold increase compared to the general population. In a long-term study of 416 patients with CVID, 38 patients were found to have malignancies, with non-Hodgkin lymphoma (NHL) representing 29 % of the total [3]. Other studies have also shown that NHL is the most frequent CVID-associated malignancy, with estimates ranging from 3 to 8 % [1, 3, 4]. However, there are far fewer reports of Hodgkin lymphoma in CVID compared to that of NHL. In one cohort of 473 patients with CVID, 32 patients (6.7 %) developed non-Hodgkin lymphoma, while there were only 4 cases (0.8 %) of Hodgkin lymphoma [5]. Data concerning the prognosis of CVID-related Hodgkin lymphoma are scarce. Among the four patients mentioned in the prior study, three developed a B-cell lymphoma years after their initial treatment, two of whom died of their lymphomas. An additional report demonstrated a poor clinical outcome in two siblings with CVID-associated Hodgkin lymphoma. Both patients died of severe sepsis 6–12 months post chemotherapy with MOPP/ABVD [6].

Rituximab, a monoclonal antibody to CD20, has been widely used in B cell NHL. Blockade of CD20 leads to impaired plasma cell differentiation. The rituximab therapy is associated with re-distribution of B cell populations to predominantly naïve cells (IgD^+^ CD27^−^) with proportional reductions in switched memory B cells (IgD^−^ CD27^+^). Transitional B cells (CD38^++^ IgM^++^) can be elevated or normal post rituximab [7]. IgG levels typically remain low 9 months after rituximab completion with impaired carbohydrate pneumococcal vaccine responses [7]. Rituximab has also been applied in the treatment for lymphocyte-predominant Hodgkin lymphoma, which are usually CD20^+^ [8]. The role of rituximab in classic Hodgkin lymphoma is less well established. Twenty to thirty percent of classic Hodgkin lymphoma expresses CD20 [9, 10]. It is speculated that rituximab would be effective in treating CD20^+^ classic Hodgkin lymphoma for a variety of reasons including the elimination of CD20-positive reactive B cells supporting Hodgkin and Reed Sternberg (HRS) cells, as well as elimination of presumptive CD20-positive HRS stem cells [11, 12]. However, clinical data are extremely limited. Brentuximab vedotin is an immunotoxin targeting CD30-expressing cells, including those in Hodgkin lymphoma [13]. It is approved for treatment of relapse/refractory Hodgkin lymphoma [14]. Ongoing clinical studies are testing its efficacy as first line treatment of Hodgkin lymphoma. Compared to conventional chemotherapy, both rituximab and brentuximab vedotin are better tolerated with less toxicity. Additionally, rituximab has been successfully used in a population of CVID patients to treat immune cytopenias [15]. Therefore these targeted agents represent attractive options for patients with CVID-associated Hodgkin lymphoma. To date, clinical data of the role of rituximab and/or brentuximab vedotin in CVID- associated Hodgkin lymphoma have not been available.

Here we report a case of a 25 year old female with CVID-associated classic Hodgkin lymphoma, who achieved complete remission with treatment of rituximab and brentuximab vedotin.

## Case presentation

A 25 year old female was diagnosed of CVID in 2006 (at age of 18) with initial presentation of recurrent episodes of bacterial sinusitis and outbreaks of genital herpes. Laboratory work revealed hypogammaglobulinemia with poor antibody responses to both polysaccharide and protein antigens. She was found to have IL-2-Inducible T-cell kinase (ITK) mutation, which is potentially involved in the pathogenesis of her CVID. She was started on IVIG and has been receiving it every month.

The patient has additional history of type 1 diabetes diagnosed at 18 months of age, for which she has been depending on insulin, as well as immune thrombocytopenia purpura (ITP) diagnosed at age of 11. She had multiple episodes of recurrent thrombocytopenia managed with IVIG and corticosteroids. In 2002, at the age of 14, she was hospitalized with a presentation of fever and pain, and found to have lymphadenopathy, lymphopenia, neutropenia, and thrombocytopenia. An extensive infectious workup was negative. She had bone marrow biopsy, which was unremarkable with the exception of findings consistent with cytopenias. She ultimately underwent a splenectomy and lymph node resection in 2002. Lymph node and spleen pathology revealed noncaseating granuloma. After the splenectomy, her fevers and pain resolved. Her blood counts have remained relatively normal since splenectomy.

**Fig. 1:**
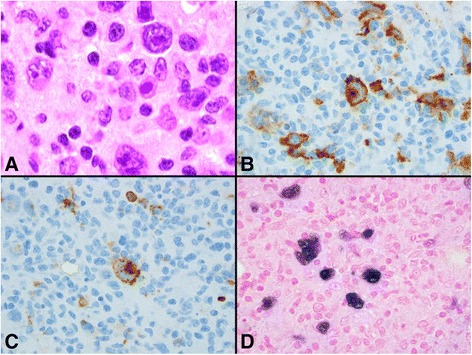
Histology of the bone marrow biopsy at the diagnosis CVID-associated classic Hodgkin lymphoma. **a** Photomicrograph of the infiltrate in the marrow comprising very large Hodgkin and Reed-Sternberg (HRS) cells in a fibroinflammatory background (Hematoxylin and eosin stain, 1000X). **b** & **c** CD30 and CD20 immunohistochemistry is positive in HRS cells respectively (3′,3′-diaminobenzidine chromogen with hematoxylin counterstain, 1000X). **d** In situ hybridization for Epstein-Barr-virus-encoded RNA 1 (EBER 1) is positive in HRS cells (INFORM EBER probe and iVIEWTMblue detection, Ventana Medical Systems, Inc., Tuscon, AZ. 1000X)

**Fig. 2:**
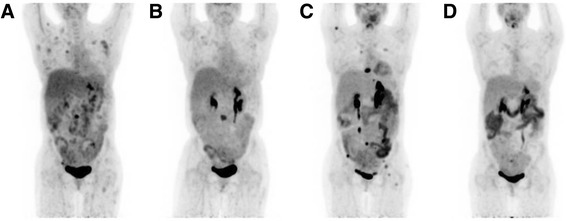
PET scan prior to and post treatment. **a** PET scan on initial diagnosis of CVID-associated classic Hodgkin lymphoma. **b** Post 4 weekly doses of rituximab. **c** Prior to brentuximab vidotin. **d** Post brentuximab

In June 2013 when she was 25 year old, she presented with left flank pain and fever. A CT scan showed diffuse lymphadenopathy in chest, abdomen, and pelvis with the largest node measuring 4.7 × 2.9 cm in the mid abdomen. A biopsy of left peri-aortic lymph node showed non-necrotizing granulomatous inflammation. Extensive infectious disease and rheumatology workup were all negative. She was started with prednisone 60 mg daily for 7 days before being tapered down, with clinical improvement. In September 2013, she was hospitalized again with abdominal pain and fever. A PET scan was done and showed extensive hypermetabolic lymphadenopathy involving the supraclavicular, bilateral axillary, right internal mammary, mediastinal, retroperitoneal, mesenteric, and pelvic lymph node chains. In addition, there were hypermetabolic sclerotic lesions within the bone marrow of L3 and the left iliac bone (Fig. 2a). She underwent left iliac bone marrow biopsy which revealed a diagnosis of EBV-positive immunodeficiency (CVID)-associated lymphoproliferative disorder, classical Hodgkin’s lymphoma morphology and immunophenotype. The abnormal cells were large cells positive for CD30, CD15, CD20 (weak to moderate), and PAX-5 (weak), EBER positive (Fig. 1). She had no other constitutional symptoms. Laboratory work demonstrated normal CBC. LDH was mildly elevated at 853 unit/L (normal range 313–618 unit/L).

Given her history of CVID, with the immunophenotype of her lymphoma, the decision was made to start therapy with rituximab monotherapy. The patient completed four weekly doses of rituximab dosed at 375 mg/m2. She had significant clinical improvement with resolution of fever and abdominal pain. LDH decreased to normal range as well. Follow-up PET/CT in January 2014 showed near-complete metabolic response in the hilar, mediastinal, axillary, retroperitoneal, mesenteric, iliac, and inguinal lymph nodes. However, there was only a partial metabolic response of the lesions in the left iliac bone, vertebral bodies T9 and L3 (Fig. 2b). Subsequently, an L3 vertebral body biopsy was pursued, with pathology consistent with the previous diagnosis of classic Hodgkin lymphoma, although with an immunophenotype that was now CD20-negative. She was referred to Radiation Oncology and received a course of 4000 cGy to the spine. She then received four additional weekly treatments of rituximab. Repeat PET/CT in June 2014 showed resolution of the L3 lesion, but revealed new sites of osseous disease in the right humerus, left posterior 5th rib, left 10th spinous process, mid sacrum, right iliac wing, and left femoral head (Fig. 2c). Brentuximab vedotin 1.8 mg/kg every 3 weeks was initiated in July 2014. The patient completed ten doses of brentuximab vedotin before discontinuing due to moderate neuropathy. She also received maintenance rituximab every 2 months. A follow-up PET/CT in December 2014 showed a complete metabolic response (Fig. 2d). Rituximab bimonthly has been maintained with good tolerance. She is doing well with no evidence of disease recurrence to date.

## Discussion

The risk of lymphoma among patients with CVID is clearly higher than that of general population. In a combined Danish and Swedish study of 176 patients with CVID, the incidence of non-Hodgkin lymphoma was 12.1-fold higher, and the incidence of Hodgkin lymphoma was 16.7-fold higher compared to that of regular population [16]. CVID-associated lymphomas are typically extra-nodal, B-cell derived and EBV negative [17]. The diagnosis of lymphoma in patients with CVID can be challenging as localized or systemic granulomatous disease and lymphoid hyperplasia are also frequently observed in this patient population [18]. As occurred in this case, initial lymph node biopsy when lymphadenopathy is discovered on body imaging is often nonspecific and nonmalignant. Repeat tissue sampling, given the chronicity of CVID, is essential to provide a timely diagnosis of malignancy in these patients.

The pathogenesis of lymphomas among patients with CVID is not well understood, but likely attributes to genetic disorder, immune dysregulation, and chronic infections. It has been postulated that the underlying immune deficient status increases the susceptibility to virus infection and the development of viral-related lymphoma (e.g. EBV associated lymphoma). In contrast to this hypothesis, most CVID-associated lymphoma is EBV negative [17]. Our patient does have EBV positive CVID-associated lymphoma. Whether the EBV status contributes to the pathogenesis of her lymphoma or it is just co-incident remains unknown. Genetic disorders in patient with CVID are heterogeneous. Our patient does have a mutation of ITK, which belongs to the Tec family of non-receptor tyrosine kinases. There are five members in Tec family: ITK, RLK (Resting lymphocyte kinase), BTK (Bruton’s tyrosine kinase), TEC (Tyrosine kinase expressed in hepatocellular carcinoma), and BMX (Bone marrow tyrosine kinase gene on chromosome X). ITK deficiency is a T-cell immunodeficiency that has been implicated in the development of EBV-positive lymphoproliferative disorders, including Hodgkins and Hodgkins-like lymphoma [19]. Our case provides the first clinic association between ITK mutation and CVID. Whether ITK plays a role in the pathogenesis of CVID-associated Hodgkin lymphoma and thus a potential target for the therapeutics of this disease is worth further studying.

Granulomatous disease is a common manifestation of CVID, occurring in 8–22 % patients [20–22]. As in our case, it is frequently noted even before the diagnosis of CVID. It has been observed that granulomatosis in CVID is associated with poor prognosis and there is no standard treatment. Most physicians choose to observation or a course of steroid if symptomatic. Long term steroid is challenging given multiple side effects including high risk of infections. Our patient did have non-caseating granulomas proved by multiple lymph node biopsies. Interestingly after initial treatment of rituximab, in addition to improvement of the metabolic active bone lesions, there was near-completed response in the extensive lymphadenopathy. Some of the lymph nodes were biopsy proven non-caseating granulomas. Therefore rituximab is potentially a promising therapeutic CVID related granulomatosis. Further study is warrantied to determine the efficacy and long term survival.

The prognosis for patients with CVID-associated Hodgkin lymphomas is unfortunately not well defined, likely due to the rareness of this disease. Our extensive literature search only located a case report in which two siblings found to have CVID-associated Hodgkin lymphoma at age of 11 and 15 respectively. However both died of severe infection 6–12 months post intensive chemotherapy [6]. One challenge is that the standard chemotherapy (e.g. ABVD) that are applied to patients with Hodgkin lymphoma who are immune competent, may not be tolerated by CVID patients because of the high risk and severity of infections resulting from immune deficiency. In our case, we chose rituximab and brentuximab instead of conventional chemotherapy as the first line treatment. The majority of classic Hodgkin lymphoma express CD30. In addition, 20 to 30 % of them express CD20 [9, 10]. Our patient did have expression of both CD20 and CD30 in her classic Hodgkin lymphoma, making a strong rationale for the treatment with rituximab and brentuximab, agents targeting CD20 and CD30 respectively. In fact, we were able to achieve complete response without major complications.

## Conclusion

In summary, we report a case of a 25 year old female diagnosed with CVID-associated classic Hodgkin lymphoma, who achieved a complete remission following treatment with rituximab followed by brentuximab vedotin. To our knowledge, our case is the first to demonstrate that rituximab and brentuximab are likely safe and effective in CVID-associated Hodgkin lymphoma, providing a feasible and potentially optimal treatment option for this patient population.

### Consent

Written informed consent was obtained from the patient for publication of this Case report and any accompanying images. A copy of the written consent is available for review by the Editor-in-Chief of this journal.
